# Entropy Metrics Correlating with Higher Residual Functioning in Patients with Chronic Disorders of Consciousness

**DOI:** 10.3390/brainsci12030332

**Published:** 2022-03-01

**Authors:** Elisa Visani, Gianvittorio Luria, Davide Sattin, Davide Rossi Sebastiano, Stefania Ferraro, Ferruccio Panzica, Matilde Leonardi, Silvana Franceschetti

**Affiliations:** 1 Fondazione IRCCS Istituto Neurologico Carlo Besta, Via Celoria 11, 20133 Milano, Italy; elisa.visani@istituto-besta.it (E.V.); luria@dima.unige.it (G.L.); davide.rossi@istituto-besta.it (D.R.S.); stefania.ferraro@istituto-besta.it (S.F.); ferruccio.panzica@istituto-besta.it (F.P.); matilde.leonardi@istituto-besta.it (M.L.); 2 Department of Mathematics, University of Genoa, Via Dodecaneso 35, 16146 Genova, Italy; 3 IRCCS Istituti Clinici Scientifici Maugeri di Milano, Via Camaldoli 64, 20138 Milano, Italy; davide.sattin@istituto-besta.it; 4 MOE Key Laboratory for Neuroinformation, School of Life Science and Technology, University of Electronic Science and Technology, Chengdu 611731, China

**Keywords:** disorders of consciousness, EEG, entropy

## Abstract

To test the ability of different entropy measures to classify patients with different conditions of chronic disorder of consciousness, we applied the Lempel–Ziv complexity, the amplitude coalition entropy (ACE), and the synchrony coalition entropy (SCE) to the EEG signals recorded in 32 patients, clinically evaluated using the coma recovery scale revised (CRS-R). All the entropy measures indicated that differences found in the theta and alpha bands can distinguish patients in a minimal consciousness state (MCS) with respect to those in a vegetative state/unresponsive wakefulness state (VS/UWS). These differences were significant comparing the entropy measure performed on the anterior region of the left hemisphere and midline region. The values of theta-alpha entropy positively correlated with those of the CRS-R scores. Among the entropy measures, ACE most often highlighted significant differences. The higher values found in MCS were for the less impaired patients, according to their CRS-R, suggest that the preservation of signal entropy on the anterior region of the dominant hemisphere correlates with better preservation of consciousness, even in chronic conditions.

## 1. Introduction

The evaluation of EEG activity in resting conditions is an important and widely applied tool in evaluating patients with disorders of consciousness (DoCs) in acute or chronic conditions (see [1] for a review). Several quantitative approaches, with uneven complexity, of EEG signal post-processing and elaboration, have been applied, including the analysis of power spectrum, complexity, entropy, or functional/effective connectivity. All these measures have the aim of suitably defining the degree of the dysfunction associated with the impaired consciousness or predicting the outcome (see recent reviews of [2,3,4,5,6]).

Several entropy estimators have been tested to quantify the “complexity” of EEG signals, mostly to assess changes associated with general anesthesia, leading to the concept that higher values could be a fingerprint of “awareness” while lowest values can be found in deeper unconsciousness. Moreover, it has been found that entropy values correlate with the Coma Recovery Scale-revised (CRS-R) [7] being lower in the Vegetative State/Unresponsive Wakefulness Syndrome (VS/UWS) than in the minimal consciousness state (MCS) and in healthy controls [8,9].

In a previous study, we evaluated the contribution of different neurophysiological tests in assessing the degree of impairment in a large case series of chronic DoC patients [10]. In this study, we aimed to validate the significance of different entropy indexes, including the Lempel–Ziv complexity, Amplitude Coalition Entropy (ACE), and Synchrony Coalition Entropy (SCE) [11] in a group of chronic DoC patients, to understand if these measures can be considered as a useful tool for obtaining information in this complex pathological condition.

## 2. Materials and Methods

### 2.1. Study Population

We consecutively included 32 adult chronic DoC patients (13 females, mean age: 50.7 ± 16.4 years; observed 32.5 ± 29.5 months after the occurrence of the acute brain damage), classified as UWS/VS (*n* = 19) or MCS (*n* = 13). Patients were observed during a week of hospitalization at the Coma Research Centre of the Fondazione IRCCS Istituto Neurologico “Carlo Besta”, Milan. Two experienced raters tested each patient independently with CRS-R (four evaluations in a week) according to the standard procedure [7,12]. Each test section was administered taking into account arousal fluctuations; every evaluation was conducted while patients were in bed (sitting position), with open eyes, far from the post-prandial time point, and without environmental interference or factors affecting and modulating brain state or patient’s activation. The median total score CRS-R value was 7.0 (range 5–16).

Patients with isoelectric or near-isoelectric EEG signals and patients with suppression burst patterns were not included. In 22 subjects, the brain damage was due to a traumatic or hemorrhagic event, while in the remaining 10 it was due to anoxic damage.

The Ethics Committee of the Institute approved the study which was performed in accordance with the Declaration of Helsinki. Legal representatives of the patients gave written informed consent for the investigation procedures.

### 2.2. EEG Recordings and Analysis

Each patient underwent a polygraph recording including EEG, EOG, ECG, spirogram, and submental EMG, which started at 2.00 p.m. on the second day after admission and lasted until 9.00 a.m. on the following day. An EEG was acquired using 19 Ag/AgCl (impedance <5 kΩ) surface electrodes, placed according to the 10–20 International System, at a sampling rate of 256 Hz (Micromed SpA, Mogliano Veneto, Italy) using a montage with a common reference electrode that allowed off-line mathematical data to be reformatted. A spline surface Laplacian estimate was applied to ensure reference-free and spatially sharpened data [13].

An artifact-free epoch lasting two minutes was selected for the analysis. To avoid possible contamination of residual EMG artifacts, mainly affecting frontopolar and temporal regions, we selected for the analyses F3, C3, P3, O1, F4, C4, P4, O1, Fz, and Cz channels, grouped in five Regions of Interest (ROIs): Left and Right FC (F3-C3 and F4-C4), Left and Right PO (P4-O2 and P3-O1) and midline (Fz-Cz). Moreover, we performed our analyses by grouping electrodes by hemisphere. We applied three distinct entropy measures: Lempel-Ziv complexity (LZc), amplitude coalition entropy (ACE), and synchrony coalition entropy (SCE), using the implementation made available by [8].

### 2.3. Statistical Analysis

We analyzed the entropy values on a wide band (1–30 Hz) and in the canonical delta (1–4 Hz), theta (4–8 Hz), alpha (8–13 Hz), and beta (13–30 Hz) bands, separately. For each band, we performed statistical analysis using repeated measure ANOVA (RMANOVA, SPSS software, version 16, SPSS Inc. Chicago, IL, USA) at a significance level of 5% using groups (MCS and VS/UWS) as between factor and ROIs or hemispheres as within-subject factors. The sphericity assumption was evaluated using Mauchley’s test, and the Greenhouse–Geisser degree of freedom correction was applied when appropriate; where the RMANOVA indicated a significant factor or interaction, post-hoc analyses by means of *t*-tests for independent or paired samples were applied, with FDR correction for multiple comparisons.

To evaluate the relationship between entropy values and CRS-R we applied linear regression analysis.

All the statistical analyses were carried out using IBM SPSS, version 20 (SPSS Inc., Chicago, IL, USA).

## 3. Results

The evaluated measures mostly gave higher values on MCS patients with respect to VS/UWS patients, in all ROIs and bands. The different entropy measures had a similar trend but SCE and LZc gave less significant statistical differences than ACE, therefore we used ACE values to prepare graphics comparing MCS and VS/UWS patients and to evaluate the relationship between the obtained results and the CRS-R values.

### 3.1. Interhemispheric Differences

RMANOVA on the hemispheres showed a significant main effect of DoC condition for ACE and LZc in theta band (F(1,30) = 4.19, *p* = 0.049, η^2^ = 0.12 and F(1,30) = 7.26, *p* = 0.011, η^2^ = 0.19, respectively), but not for SCE. We found no main effects of the etiology of the injury, whether classified as anoxic vs hemorrhagic or traumatic.

**Figure 1:**
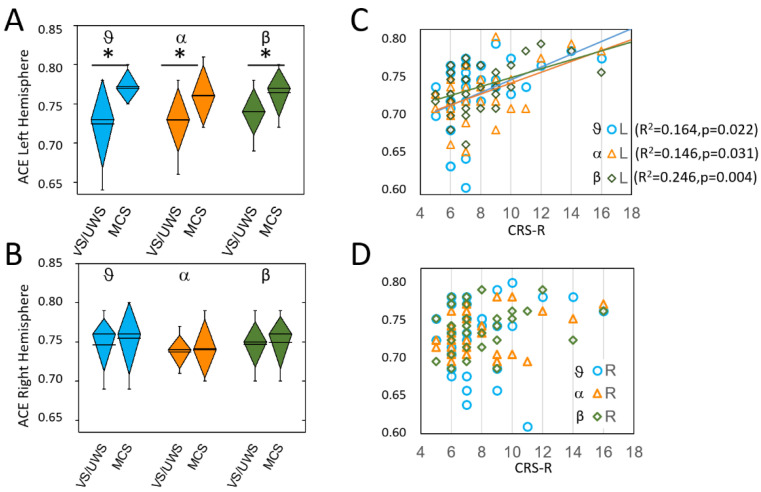
ACE values on left (L) (**A**); and right (R) (**B**) hemispheres. Significant differences between MCS and VS/UWS were observed on the left hemisphere only and are marked with asterisks. In each diamond box, the line represents the mean value, the diamond height represents the SD and the whiskers represent the 10–90% range of the values. Panels C and D show, with the same colors as in A and B, the linear relationships between ACE measures and CRS-R score, which were significant on the left hemisphere only (the lines are shown only for significant relationships, with the same color of the symbols). The theta values are in light blue (circles in (**C**,**D**)), the alpha values in orange (triangles in (**C**,**D**)), and the beta values are in dark green (diamonds in (**C**,**D**)).

The post-hoc comparisons indicated that the MCS patients, when compared with VS/UWS patients, had significantly higher values on the left hemisphere for all frequency bands including wide band (Table 1, Figure 1A). We found no difference for any of the entropy measurements on the right hemisphere between the MCS and VS/UWS patients (Figure 1B).

Paired *t*-test for interhemispheric difference showed for MCS patients, but not for VS/UWS patients, significant differences with higher values on the left hemisphere in theta (MCS: t(12) = −2.6, *p* = 0.021; VS/UWS: t(18) = 0.6, *p* = 0.572, alpha (MCS: t(18) = −2.7 *p* = 0.019; VS/UWS: t(18) = 1.4, *p* = 0.163) and beta (t(12) = −2.6, *p* = 0.024; VS/UWS: t(18) = 0.9, *p* = 0.384) bands.

Linear regression analysis applied to different bands between ACE values and CRS-R scores showed on the left hemisphere a significant direct relationship in theta (F(1,30) = 5.89. *p* = 0.022. R^2^ = 0.164), alpha (F(1,30) = 5.13. *p* = 0.031. R^2^ = 0.146) and beta (F(1,30) = 9.78. *p* = 0.004. R^2^ = 0.246) bands (Figure 1C). No significant relationships were found for the right hemisphere (Figure 1D).

### 3.2. Selected ROIs

RMANOVA indicated a significant main effect of the DoC condition for ACE in the theta and alpha bands (F(1,30) = 5.97, *p* = 0.021, η^2^ = 0.17 and F(1,30) = 4.47, *p* = 0.043, η^2^ = 0.13, respectively) and for the LZc values for the alpha band (F(1,30) = 6.72, *p* = 0.015, η^2^ = 0.18), but not for SCE values in any band. We found no main effects of the etiology of the injury, whether classified as anoxic vs hemorrhagic or traumatic.

Post-hoc comparisons with FDR correction for multiple comparisons were reported in Table 1.

**Figure 2:**
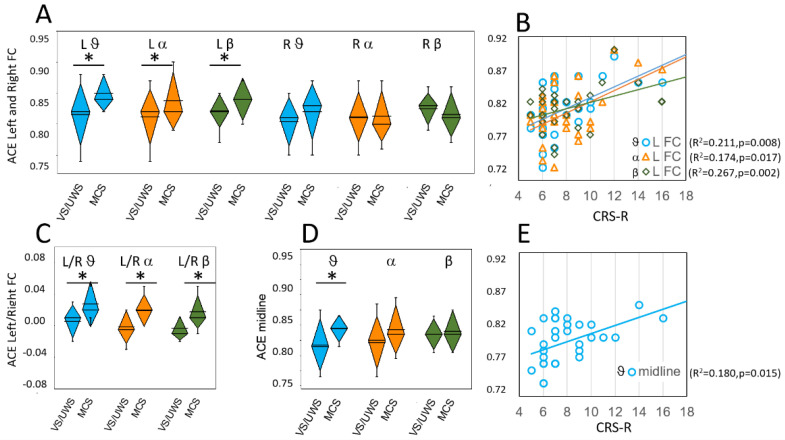
ACE values on left (L) and right (R) fronto-central (**A**); and midline (**D**) regions. Panels (**C**) shows the ratio between measures on left and right fronto-central regions; Panel (**B**) shows the linear relationships between ACE values obtained on the left fronto-central region and CRS-R values; Panel (**E**) shows the linear relationship between ACE values on the midline region and CRS-R values. Values and lines that did not give significant relationships are not shown. Colors and symbols as in Figure 1. Significant differences are marked with asterisks.

The ACE values were significantly higher in MCS patients on the left FC ROI in theta (t(30) = −2.9, *p* = 0.007) and in alpha bands (t(30) = −3.0, *p* = 0.005) and at the significance limits for beta bands (t(30) = −2.6, *p* = 0.014) (Table 1, Figure 2A).

Paired *t*-test between left FC and right FC ROIs indicated a significant difference in MCS patients (theta: t(12) = −2.9, *p* = 0.014; alpha: t(12) = −3.6, *p* = 0.004 and beta: t(12) = −3.6, *p* = 0.003), but not in VS/UWS patients (theta: t(18) = −1.5, *p* = 0.159; alpha: t(18) = 0.9, *p* = 0.344 and beta: t(18) = −0.0, *p* = 0.981). Figure 2C shows the ratio measured between left and right FC ROIs in VS/UWS and MCS patients.

Linear regression analysis performed on ACE and CRS-R values revealed a significant direct relationship only for the left FC region (Figure 2B) in theta (F(1,30) = 8.00, *p* = 0.008, R^2^ = 0.211), alpha (F(1,30) = 6.33, *p* = 0.017, R^2^ = 0.174) and beta band (F(1,30) = 10.93, *p* = 0.002, R^2^ = 0.267). We did not find significant relationships in the 1–30 Hz band or the delta band. Linear regression showed no significant relationships in any frequency band for both the right FC region and the right and left PO regions.

On the midline region, the ACE values were significantly higher in MCS patients than in VS/UWS patients on the theta band only (t(28.7) = −2.58, *p* = 0.015) (Figure 2D). Regression analysis showed a significant relationship between ACE values and CRS-R values in the theta band (F(1.30) = 6.60, *p* = 0.015 R^2^ = 0.180) (Figure 2E).

## 4. Discussion

We designed the present study to evaluate whether different entropy measures may be useful in providing information on the degree of severity in patients with chronic DoCs. Therefore, we did not compare DoC patients with a control population, keeping also in mind that the general severity of brain damage in the evaluated subjects raises doubt about the value of the comparison with a normal EEG.

Several previous studies investigated the EEG of DoC patients in resting-state conditions with entropic measures to study signal complexity and its significance [3,14,15,16,17,18] assuming brain activity can manifest under resting conditions, without needing specific types of stimuli protocols [19]. Specifically, the entropy ACE or SCE measures, which we applied, were previously validated in determining the “consciousness” changes in case of anesthesia [11], drug-induced psychedelic state [20], or sleep [21,22] but not, to our knowledge, to patients with chronic DoCs. We found that all the three applied entropy measures had a similar trend, but ACE values more often returned significant differences. The same occurred when evaluating the relationship between the entropy values and CRS-R ones using linear regression analysis.

In the delta band, we observed higher ACE values in MCS patients than in VS/UWS patients only when comparing the values measured on the entire left hemisphere, but, in no case, for the single ROIs. The limited relationship between delta entropy values and the severity of DoC is in agreement with the observation of a lack of relationship between the ACE values in the delta band with the depth of Propofol anesthesia [11].

Our main finding indicates that MCS patients compared with VS/UWS patients had significantly higher ACE values in theta, alpha, and, to a lesser extent, beta bands, and this prominently involved the frontocentral region of the left hemisphere. Moreover, MCS patients had higher ACE values in the theta band, also on the midline region. This evidence may suggest that a “regional” complexity of alpha/theta activity plays a pivotal role in distinguishing between higher (MCS) and lower (VS/UWS) residual cortical functioning. Various previous evidence conversely supports the main role of the posterior cortical regions, which includes sensory areas, in consciousness’ preservation [23]. Our evidence did not necessarily contrast this possibility when comparing DoC patients with healthy subjects. We compared only patients in different DoC conditions, thus our data simply suggest that in a population of DoC patients the anterior (fronto-central) region of the dominant hemisphere may play a main role in consciousness’ preservation.

The prominent ACE values on the left front-central region may perhaps reflect better functional preservation of the dominant hemisphere in MCS patients compared with VS/UWS patients. In patients with DoCs, the role of the dominant hemisphere has been little explored through EEG analysis techniques, therefore the higher ACE values that we found on the left FC region may be considered as a novel finding. The role in consciousness preservation of the dominant hemisphere has been highlighted in other pathological conditions. Detyniecki et al. [24] found that loss of consciousness due to epileptic seizures is more common and worse in patients when the ictal discharge begins on the left hemisphere; moreover, some reports suggested that loss of consciousness occurs more commonly in patients with left hemispheric stroke [25].

Higher ACE alpha values on the left anterior region that we found in less severely affected patients may meet the value of frontal alpha rhythm asymmetry observer in several conditions and be considered as a psychological and neural index of different pathological neuropsychological conditions (for a review, see [26]). Hence, we can hypothesize that high EEG entropy in the alpha band on the fronto-central region of the left (dominant) hemisphere may support higher residual cognitive functions.

In this study, which we consider a pilot exploration of new entropy indices in patients with DoC, we applied the most widely used method in assessing impaired consciousness, based on CRS-R values. Certainly, we did not specifically explore specific residual functions that possibly derive from better functional preservation of the dominant hemisphere. We consider that we can extend the entropy measures, in particular the ACE measure, in a more extensive series by evaluating specific functions and recently proposed measures to assess the level of consciousness’ impairment [27].

Higher ACE theta values were found in MCS patients not only on the left FC region but also on the midline region, with a significant linear relationship with CRS-R scores. This can be in line with previous evidence obtained in MCS patients compared with VS/UWS during long-lasting recordings by [28], proposing that some patients have higher spectral entropy in the theta-alpha band on the midline region, but a substantial time variability reflects on the inconsistency of cognitively mediated behaviors.

Some limitations of this study must be accounted for, including the restricted number of EEG channels in our recordings, which limits precise topological considerations, the relatively limited number of evaluated patients, and the application of clinical scales, limited to the CRS-R, with no exploration of specific residual brain functions. To improve our results in the future we will apply other scores such as the Modified Score or the CRS-R index in association with our entropy analysis in a larger population.

## 5. Conclusions

Our results may offer a new concern about the functional re-organization occurring after a brain lesion using entropic indices, even if the data presented certainly require verification in a more extensive case series and further evaluation by means of other indices of consciousness or specific brain functions impairment. However, the applied entropy measures do not require complicated post-processing and can be expected to help in the evaluation of DoC patients, thus potentially becoming promising for bedside observation in chronic conditions.

## Figures and Tables

**Table 1:** Post hoc comparison of entropy measures assessed in MCS vs. VS/UWS patients in different regions (*) indicates value not surviving FDR correction).

Region	Frequency Band	VS/UWS vs MCS ACE	VS/UWS vs MCS SCE	VS/UWS vs MCS LZc
		t(df), *p*	t(df), *p*	t(df), *p*
Left Hemisphere	1–30 Hz	t(26.9) = −3.8, *p* = 0.001	t(22.8) = −3.0, *p* = 0.006	t(30) = −2.1, *p* = 0.047 *
	1–4 Hz	t(26.6) = −2.7, *p* = 0.012	t(23.6) = −2.3, *p* = 0.029 *	t(29.3) = −2.8, *p* = 0.009
	4–8 Hz	t(25.4) = −3.3, *p* = 0.003	t(23.9) = −2.8, *p* = 0.010	t(25.8) = −3.2, *p* = 0.003
	8–13 Hz	t(30) = −2.8, *p* = 0.010	t(30) = −2.0, *p* = 0.051	t(30) = −1.8, *p* = 0.084
	13–30 Hz	t(30) = −2.3, *p* = 0.026 *	t(30) = −1.8, *p* = 0.075	t(30) = −1.1, *p* = 0.275
Right Hemisphere	1–30 Hz	t(30) = −0.7, *p* = 0.504	t(30) = −0.5, *p* = 0.621	t(30) = −1.6, *p* = 0.114
	1–4 Hz	t(30) = −0.4, *p* = 0.676	t(30) = −0.8, *p* = 0.408	t(30) = 0.1, *p* = 0.958
	4–8 Hz	t(30) = −1.0, *p* = 0.334	t(30) = −0.8, *p* = 0.399	t(30) = −0.9, *p* = 0.374
	8–13 Hz	t(17.1) = −0.3, *p* = 0.371	t(18.2) = 0.4, *p* = 0.697	t(17.2) = −1.2, *p* = 0.159
	13–30 Hz	t(30) = −0.1, *p* = 0.909	t(30) = −0.9, *p* = 0.379	t(30) = −0.7, *p* = 0.458
Midline	1–30 Hz	t(30) = −2.0, *p* = 0.051	t(30) = −1.4, *p* = 0.179	t(30) = −2.1, *p* = 0.041 *
	1–4 Hz	t(30) = −1.4, *p* = 0.161	t(30) = −1.0, *p* = 0.307	t(30) = −1.1, *p* = 0.287
	4–8 Hz	t(28.4) = −2.7, *p* = 0.011	t(30) = −1.9, *p* = 0.063	t(27.6) = −3.60, *p* = 0.001
	8–13 Hz	t(30) = −1.4, *p* = 0.166	t(30) = −1.3, *p* = 0.188	t(30) = −2.4, *p* = 0.022
	13–30 Hz	t(30) = −0.4, *p* = 0.665	t(30) = −0.5, *p* = 0.632	t(30) = −0.5, *p* = 0.631
Left fronto-central	1–30 Hz	t(30) = −2.4, *p* = 0.025 *	t(30) = −1.6, *p* = 0.115	t(30) = −1.9, *p* = 0.069
	1–4 Hz	t(30) = −1.2, *p* = 0.259	t(28.1) = −0.8, *p* = 0.424	t(30) = 0.1, *p* = 0.876
	4–8 Hz	t(30) = −2.9, *p* = 0.007	t(30) = −2.4, *p* = 0.021 *	t(27.8) = −2.1, *p* = 0.045 *
	8–13 Hz	t(30) = −3.0, *p* = 0.005	t(30) = −2.2, *p* = 0.039 *	t(30) = −2.1, *p* = 0.042 *
	13–30 Hz	t(30) = −2.6, *p* = 0.014 *	t(30) = −1.5, *p* = 0.131	t(30) = −0.5, *p* = 0.598
Right fronto-central	1–30 Hz	t(30) = −0.5, *p* = 0.597	t(30) = −0.3, *p* = 0.802	t(30) = −1.9, *p* = 0.069
	1–4 Hz	t(30) = −0.3, *p* = 0.744	t(30) = −0.6, *p* = 0.536	t(30) = 0.8, *p* = 0.453
	4–8 Hz	t(30) = −1.3, *p* = 0.208	t(30) = −0.8, *p* = 0.426	t(30) = −1.3, *p* = 0.214
	8–13 Hz	t(30) = −0.2, *p* = 0.827	t(30) = −0.1, *p* = 0.960	t(30) = −1.8, *p* = 0.073
	13–30 Hz	t(30) = 0.7, *p* = 0.468	t(30) = 1.0, *p* = 0.298	t(30) = −0.4, *p* = 0.715
Left parieto-occipital	1–30 Hz	t(29.6) = −1.9, *p* = 0.071	t(30) = −1.5, *p* = 0.139	t(30) = −1.7, *p* = 0.105
	1–4 Hz	t(30) = −1.9, *p* = 0.064	t(30) = −1.7, *p* = 0.101	t(30) = −1.4, *p* = 0.157
	4–8 Hz	t(30) = −1.4, *p* = 0.182	t(30) = −1.2, *p* = 0.236	t(30) = −1.5, *p* = 0.188
	8–13 Hz	t(30) = −0.5, *p* = 0.619	t(30) = −0.2, *p* = 0.842	t(30) = −0.7, *p* = 0.470
	13–30 Hz	t(30) = −1.8, *p* = 0.089	t(30) = −0.9, *p* = 0.374	t(30) = −0.3, *p* = 0.723
Right parieto-occipital	1–30 Hz	t(30) = −0.7, *p* = 0.487	t(30) = −0.7, *p* = 0.504	t(30) = −1.5, *p* = 0.145
	1–4 Hz	t(30) = −0.4, *p* = 0.702	t(30) = −0.9, *p* = 0.366	t(30) = 0.9, *p* = 0.376
	4–8 Hz	t(30) = −0.8, *p* = 0.448	t(30) = −0.6, *p* = 0.536	t(30) = −1.3, *p* = 0.215
	8–13 Hz	t(30) = −0.7, *p* = 0.501	t(30) = 0.5, *p* = 0.597	t(30) = −1.7, *p* = 0.098
	13–30 Hz	t(30) = −0.9, *p* = 0.368	t(30) = 0.1, *p* = 0.960	t(30) = −0.8, *p* = 0.430

## Data Availability

All custom scripts and data contained in this manuscript are available upon request from the corresponding author.
